# The quantum brain: one psychology

**DOI:** 10.3389/fpsyg.2025.1660500

**Published:** 2025-12-15

**Authors:** Elif Zapsu

**Affiliations:** Department of Psychology, Üsküdar University, Istanbul, Türkiye

**Keywords:** quantum brain, non-duality, psychotherapy, holonomic brain theory, spirituality

*The unexpected parallelisms of ideas in psychology and physics suggest…a possible ultimate oneness of both fields of reality that physics and psychology study…The concept of a unitarian idea of reality…was called by Jung the* ***unus mundus***. (von Franz, 1964, p. 309)

The questions of who and what we are consistently emerge at the center of humanity, with some looking to science and religion for answers. Politics appears to impact ones ability to understand oneself truly. As one source, we create endless unique attribute combinations: intellect, love, wisdom, strength; yet, the names and labels assigned to this diversity have created a sense of separation. This may, in turn, generate power relationships and give rise to politics. Perhaps, to exist is to be political. Nonetheless, it is worth considering what might occur if there were a non-dual perspective on the brain—one that embraces all as itself without being constrained by religion or place. Indeed, there will always be differences in power, as is the beauty of existence; however, by further experiencing essential unity and unconditional acceptance, one might achieve greater peace, freedom, and knowledge of the *true self* .

Therefore, this paper aims to propose a unique perspective on the quantum brain and to foster research and clarity on the fundamental physics underlying human existence within the context of psychology. It seeks to further the dialogue on understanding the post-material perspective of the brain, the constructed identity of the self, and the potential implications for the field of psychology and psychological therapies.

## The physics of non-duality

Although this author writes from a very limited perspective of a psychologist with no formal higher education in physics, one must have courage and attempt to decipher, even with a childlike comprehension, the remarkable findings and works of great minds across disciplines. Physics is the study of the mechanics of the universe, ranging from the smallest to the largest scales and from the most unseen to the most perceivable (Susskind and Friedman, 2014, p. xix). Quantum theory is used to explain the micro in our macro universe (Aerts, 2014), the most elegant mystery of existence. Our quantum existence depicts a field-like energetic unit in which all matter is 99.9% empty and consists solely of information (Rutherford, 1911; Wheeler, 2002, p. 314). This statement is enough to spend ones life contemplating and has incredible implications for a frequency-based psychological interpretation of the world.

In 1918, Max Planck won a Nobel Prize (in Nobel Prize, 2025a) for the discovery of energy “quanta.” Subsequently, the field of Quantum Physics has undergone extraordinary developments. Currently, numerous astounding physics discoveries have led to the conclusion that the basic structure of the cosmos requires observers to shape its existence (Lanza et al., 2020, p. 6). Research has found that the principle of non-locality (Bohm, 1990, p. 274) demonstrates how one might transcend time and space, while the principle of entanglement (Bornman, 2021) depicts how, despite large distances, two particles can “act” as one, potentially describing the process of “empathy.” Increasingly, research appears to depict an eternal, non-local unity, a oneness, where

“the world cannot be analyzed correctly into distinct parts; instead, it must be regarded as an indivisible unit ….” (Bohm, 1979, p. 161)

Remarkably, some argue that no single quantum theory prediction has been proven wrong (Rosenblum and Kuttner, 2011, p. 5).

The theory of the holographic principle, as proposed by Nobel laureate t'Hooft (2001), expanded upon and currently taught by physicist Susskind (1995) at Stanford University, suggests that the entire universe, specifically our world and ourselves, is holographic in nature—a metaphor for complex mathematics. This study is based on the scientific models developed by Maldacena (1999), following his work on black holes. The data (content) of black holes is equivalent to their surfaces, using a function akin to a holographic conversion (Maldacena, 1999). Accordingly, albeit a significant simplification, if mathematics suggests that the universe is a projection, then our bodies would be akin to avatars, a reflection of data in a single unit of nothingness—a point beyond space-time. These findings have several important implications. It is possible that the true self may be the observer of this holographic body, residing beyond space-time as endless data, and one might be able to shift the point of observation from the holographic body to become an observer of all forms from a nonlocal perspective. These are incredible times for scientific discoveries; nevertheless, despite these scientific advancements, Dr. Richard Feynman, winner of the Nobel Prize in Physics in 1965 (Nobel Prize, 2025b), stated that no one can truly understand quantum mechanics due to its inherent complexity (BBC Archive, 2018). With such limitations of science in mind, this author suggests that students of psychology should learn these core aspects of the nonmaterial world with greater clarity and attempt to make sense of them in reference to understanding the brain, to the best of their ability.

“*The part mirrors the whole*.” (Muhammad saw in Hulusi, 2012a, p. 7)

## The unitary brain

**Figure 1:**
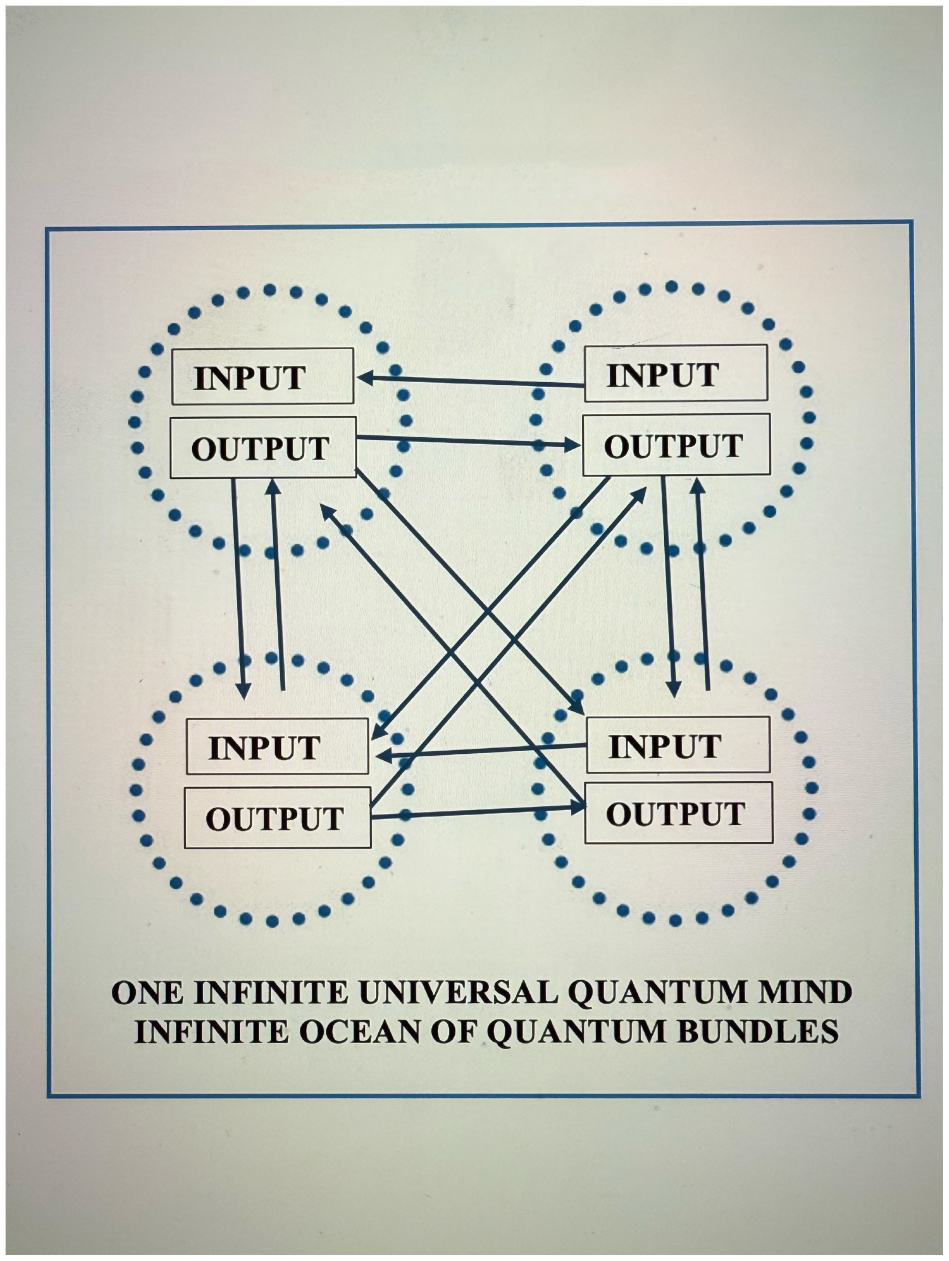
A non-dual quantum bundle, a holonomic biocomputer.

One might hypothesize that the concepts discussed above are also applicable to the brain from a quantum perspective. Although quantum cognition is a controversial approach to understanding consciousness, increasing evidence supports its theoretical models. Authors have written extensively about the quantum brain in academic research, in powerful ways (Jibu and Yasue, 1995; Tarlaci, 2025). Evidence-based research suggests that quantum entanglement processes are involved in neural networks, supporting quantum brain models (Liu et al., 2024). Bohm's work (Bohm, 1990, p. 273) on the implicit and explicit realms indicates the non-duality of what this author has termed “quantum bundles” (Figure 1) and the brain's function as a holonomic whole. Here, the author reflects on the brain as a “holonomic biocomputer,” functioning as an (input–output) computer system (Pribram, 1975, p. 164), working as a whole unit, with each part processing the entire organ, and where all *Its (particles, parts) derives its function from bits* (Wheeler, 2002, p. 310). This perspective can be supported by recent research on microtubules in neurons as information carriers (Hameroff, 2022; Babcock et al., 2024). These create a holomovement in which consciousness and matter are one, and in which neurons act as a single whole—the macro-neuron—utilizing the principle of nonlocality (Bohm, 1990, p. 274). This process opposes the traditional Cartesian *dualistic understanding of the interaction* between mind and body and instead suggests a non-dual perspective in which the mind is the body and the body is the mind, although the mind-body relationship can continue eternally (e.g., spirit-body).

One of the most comprehensive approaches to the quantum mind is the Bohm-Penrose-Hameroff model of consciousness (Gallego, 2011), a synthesis of quantum concepts, though it neglects the valuable work of Pribram (1975), perhaps indicating a gap in the literature of integrative sciences in relation to psychology. This author argues for a psychological perspective where one is in coherent unity through the holonomic brain (microunit) with the holographic universe (macrounit). One must note, for further clarification, that the prospect of a nonmaterial, holonomic brain does not negate its very real biological mechanisms, in which the system works as a whole—from physics and chemistry to biology and psychology—from quantum biopsychology to the invisible-visible.

## To be or not to be?

If one is not confined to this body and all the labels it contains, and the body is our holonomic avatar, then all is one in essence, and the so-called ego identity, or self-construct, may merely be a collection of conditions, value judgments, and emotions that claim a false and illusory sense of self (Hulusi, 2016, p. 9). As cognitive psychology and neuroscience might contest, the ego-based self is “illusory”—a controlled hallucination unique to each individual (Hoffman, 2019; Seth, 2022). Here, one must reiterate that religious and spiritual perspectives may include the belief in a spirit “form” or avatar that continues eternally, as “the holographic-quantum brain harbors data and lives eternally. It creates the illusion of existence based on that data” (Hulusi, 2020, p. 74).

With a greater understanding of our “true selves,” suffering could decrease drastically, as the experience of peace and love increases. One would view the self as essentially one, as ones identity would no longer be based on an “illusory” concept, traditionally referred to as the ego (Zapsu, 2023, p. 245). One can then decondition the False Self, which creates a sense of duality (Hulusi, 2012b, p. 78) to achieve a peaceful state of unity and spiritual actualization.

It is time for this topic to be openly discussed within the field of Psychology. Further research and public awareness in this area are necessary. This study has limitations and questions that require attention. How exactly does the brain function in terms of quantum mechanics? How can one develop a psychology of physics course that is internationally accepted by academia? What are the limitations of these findings? Why is it taboo to merge elementary physics with psychology, but not with other disciplines such as biology, philosophy, history, the arts, and business? Can a *universal spiritual* framework be used for the quantum brain? This author suggests that there is much to gain by avoiding fear and embracing change. Scientific research indicates that our existence and the universe are undoubtedly one indivisible unit (Päs, 2023), arguably one consciousness. It is time for us to respect these findings and position ourselves accordingly.

## Bienvenue à la renaissance

It is possible that believing that one is not confined to the body and its labels (conditions, value judgments, and emotions) can be healing and beneficial (Zapsu, 2023). Additionally, it can be suggested that the more one understands how the holonomic brain functions, the more one learns what it means to be human. There may be sufficient knowledge of holonomic theory to initiate trials of the efficacy of quantum brain models in the psychotherapeutic realm. Hopefully, this field will yield positive results, including greater peace, unity, spiritual actualization, and empowerment.

## Author's note

Limitations of language, such as “projection” and “reflection”, can falsely suggest duality.
